# Improved Sphincter Muscle Regeneration by Myoblasts from *M. extensor carpi radialis* in a Large Animal Model of Urinary Incontinence

**DOI:** 10.3390/medsci14010027

**Published:** 2026-01-06

**Authors:** Niklas Harland, Lukas Schwarz, Meltem Avci-Adali, Andrea Buzanich-Ladinig, Lina M. Serna-Higuita, Arnulf Stenzl, Wilhelm K. Aicher

**Affiliations:** 1 Centre for Medical Research, University of Tuebingen Hospital, 72072 Tuebingen, Germany; 2 Clinical Centre for Population Medicine in Fish, Pig and Poultry, Clinical Department for Farm Animals and Food System Science, University of Veterinary Medicine Vienna, 1210 Vienna, Austria; 3 Department of Thoracic and Cardiovascular Surgery, University of Tuebingen Hospital, 72076 Tuebingen, Germany; 4 Institute for Clinical Epidemiology and Applied Biometry, University of Tuebingen Hospital, 72076 Tuebingen, Germany; lina.serna-higuita@med.uni-tuebingen.de

**Keywords:** stress urinary incontinence, large animal model of incontinence, cell therapy, sphincter regeneration, myoblasts

## Abstract

**Purpose:** Stress urinary incontinence (SUI) is a significant medical challenge affecting substantial parts of modern societies. Several studies suggested that cell therapy may alleviate the symptoms. However, in many cases, the overall efficacy was not satisfactory for the patient’s needs. Moreover, in our recent preclinical studies, myoblasts isolated from *M. semitendinosus* failed to restore significant urethral sphincter function. We, therefore, investigated in our large animal SUI model whether myoblasts from other muscles yielded better sphincter recovery. **Methods:** Urethral sphincter deficiency was induced surgically in six female littermates and confirmed by measuring the urethral wall pressure. Three days after induction of sphincter deficiency in gilts, homologous myoblasts were injected into the sphincter complex. The urethral wall pressure and urine status were monitored weekly for a six-week follow-up. **Results:** Myoblasts isolated from *M. extensor carpi radialis* yielded a high expression of the myogenic markers desmin, CD56, ACTA1, MSTN, Myf6, and MyoD; were differentiation-competent; and formed myotubes in vitro. Such cells restored significant sphincter deficiency (2494 ± 266 U; ≙92%; *p* < 0.001; n = 6) and yielded a complete functional recovery from the induced sphincter deficiency (481 ± 123 U, ≙18%) when compared to the starting levels of untreated healthy pigs (2683 ± 764 U; ≙100%). The experimental group showed significant recovery compared to the mock controls (*p* < 0.045). **Conclusions:** The choice of myoblasts contributes to the clinical outcome in our large animal model of urinary incontinence. Myoblasts from *M. extensor carpi radialis* facilitated better sphincter recovery compared to myoblasts from *M. semitendinosus*.

## 1. Introduction

Stress urinary incontinence (SUI) is an increasing challenge for many modern societies as its incidence rises among the elderly in ageing populations [1,2,3,4,5]. Medical records and patient surveys suggest that about 37% of elderly women suffer from any form of urinary incontinence (UI) [2,6], while the prevalence in men can reach up to 30% [5]. Incontinence is linked with various secondary conditions, including social isolation, recurrent urinary tract infections, and dermatitis [7,8,9]. Individuals experiencing mild UI may manage the condition by adjusting fluid intake and output. Physical exercise and medication can help alleviate the symptoms [7,10]. In severe cases of SUI, surgical interventions may be necessary to achieve satisfactory results [7].

SUI was linked to a loss of functional muscle tissue, urethral scar formation, and impaired innervation [7,11]. This has led to the idea that improving sphincter muscle function could be achieved by locally applying active components that promote tissue regeneration, including adipose tissue-derived mesenchymal stromal cells (ADSCs) and myogenic progenitor cells (MPCs) [12]. Several preclinical studies have indicated that cell therapies might improve incontinence [13]. Similarly, some clinical studies have reported success [14,15,16,17,18]. Others, however, failed to show convincing results [19]. The double-blind ADMC-USR study reported placebo responses [20]. Nevertheless, cell-based SUI therapies demonstrated high cost-effectiveness [21]. However, the current evidence and situation remain unsatisfactory for the field. 

The partly contradictory reports may be due to technical differences between the studies. One challenge is the application of active components in the region of interest [22,23,24]. The quality of injected cells may also influence the outcomes of SUI therapies. In our recent study, ADSCs demonstrated a significant recovery in urethral wall pressure in a porcine large animal model of SUI cell therapy [25]. In contrast, MPCs derived from *M. semitendinosus* did not produce a full and significant recovery [25]. Furthermore, in clinical studies of SUI cell therapy, myoblasts from various muscles were used, likely following different protocols [11,14,17,18,26,27], and most of these studies did not characterise the myoblasts in detail. In this context, it gains importance that different porcine muscles contain quite distinct numbers of myogenic progenitor cells [28]. Thus, we hypothesised that the number and quality of the MPCs applied for sphincter regeneration might play a role in the regeneration of the deficient urinary closure apparatus in our model. Therefore, we expanded our research to investigate, in an exploratory study, whether MPCs isolated from other striated muscles might offer a better outcome in SUI cell therapy within this model.

## 2. Materials and Methods

### 2.1. Production of Myogenic Progenitor Cells

A 5-day-old male landrace littermate of the gilts to be employed in the SUI cell therapy study was anaesthetised (10 mg/kg body weight (BW) ketamine, 0.5 mg/kg BW azaperone i.v.) and sacrificed. The death of the boar was confirmed via auscultation of absent heartbeat noises and testing the corneal reflex. Based on the amounts of satellite cells and MPCs in different muscles [28], tissue samples of *M. extensor carpi radialis (Mecr)*, *M. rhomboideus cervicis (Mrc)*, and *M. fibularis tertius (Mft)* were isolated aseptically. The MPCs were prepared as described [28,29]. The tissue samples were minced and subjected to proteolytic digestion (HBSS, 0.2% collagenase 1, 0.01% DNase 1, 0.025% trypsin). The cells were enriched by a cell strainer, centrifuged (800× *g*, 10 min. 4 °C), purified by a Percoll gradient (15,000× *g*, 10 min; Sigma-Aldrich, Taufkirchen, Germany), washed, and expanded (=bath 1 MPCs). Cells not released by proteolysis from the minced tissue were collected and expanded in a separate lot (=batch 2 MPCs).

The cells were expanded in expansion medium (E-medium: F10 medium, 15% FBS, 5 ng/mL bFGF, antibiotics, all from Sigma-Aldrich) [28,29]. The cell proliferation rate was monitored, and the MPCs were characterised. Differentiation and myotube formation were induced by differentiation medium (DMEM, low glucose, 2% horse serum, antibiotics) and documented by brightfield microscopy and immunofluorescence.

### 2.2. Analysis of Transcript Expression

The mRNA expression of myogenic marker genes was enumerated by RT-PCR [29,30]. The RNA was isolated (Aurum Total RNA mini kit; BioRad, Dreieich, Germany), yield and purity were determined by UV spectroscopy (Eon BioTec Instruments, Hillborough, NC, USA), and 1 μg was transcribed reversely (iScript cDNA synthesis kit; BioRad). For amplification of target genes and the housekeeping reference GAPDH (Table 1), a hot-start protocol followed by PCR amplification (39 cycles; iQ SYBR Green Supermix chemistry (BioRad)) and melting point analysis was performed (CFX95 Real-Time System; BioRad). The expression of the myogenic marker genes was normalised to the expression of GAPDH by the 2^(−∆∆CT)^-method [31,32] as described by the manufacturer (CFX Maestro software V2.3, BioRad).

### 2.3. Immunocytochemistry of Myoblasts

The expression of myogenic markers was explored by immunocytochemistry (ICC) [33]. The MPCs were washed with PBS, fixed (4% paraformaldehyde), washed, permeabilised (0.1% Triton X-100 in PBS (=T-PBS), and washed again with PBS. The cells were pre-incubated with blocking solution (5% dry milk powder). Desmin was detected by a rabbit antibody (# 15200, 1:200, abcam, Cambridge, UK) and a detection antibody (FITC- or PE-labelled F(ab’)2-donkey-anti-rabbit IgG, #711-096-152, 1:200, Jackson-Dianova, Hamburg, Germany). Unbound antibodies were aspirated, and the samples were washed with PBS-T. F-actin was detected by phalloidinstaining (ReadyProbes, Invitrogen, Carlsbad, CA, USA) and CD56 was detected by PE-labelled anti-CD56 antibody (clone MEM-188, 1:200, BioLegend, San Diego, CA, USA). The cell nuclei were counterstained by DAPI (Sigma-Aldrich). Samples omitting the primary reagents served as controls. The marker expression was observed by fluorescence microscopy (BZ-X800, Keyence, Mechelen, Belgium), evaluated by proprietary software (BZ-H1ase, Keyence). To compare the staining intensity of desmin on the different MPCs, the desmin- and DAPI-stained micrographs were each converted to an 8-bit grey-scale image (Atkinson mode; ImageJ V1.54g, NIH), and the positive signals were enumerated to compute a desmin staining intensity index, normalised to DAPI.

### 2.4. Flow Cytometry of Myoblasts

The expression CD56 was quantified by flow cytometry (FC) [34,35,36]. The MPCs were detached, washed, and counted, and 5 × 10^5^ MPCs were resuspended in FC sample buffer and incubated with PE-labelled anti-CD56 antibody (NCAM, # 318305, 1:10, BioLegend). The cells were washed, resuspended in 300 µL FC sample buffer, and analysed by FC (FACSCelesta; BD Biosciences, Heidelberg, Germany). Cells without antibody staining and FC compensation particles (BD Bioscience) were used to set the gates. The size and granularity of the MPCs were determined by the forward (FSC-A) and sideward (SSC-A) scatters, respectively. Debris and dead cells were excluded, and the expression of CD56 was enumerated. The data were processed using proprietary software (FACSDiva V9.0, FlowJo10.7.1; BD Biosciences).

### 2.5. Pig Husbandry and Providing a Large Animal Model of Urinary Incontinence

In our recent studies, cohort sizes of five pigs were computed to yield significant results in the pig SUI cell therapy model [25,37]: In this study, the sample size estimation was preformed using a two-group repeated measures design with an intra-subject correlation of 0.5 between repeated measurements. A sample size of five animals per group was required to achieve 80% to detect a mean difference of 80 mm H_2_O by a urodynamics device (see below) [38] at a two-side significance level of α = 0.05, assuming a correlation of 0.5a. To account for potential attrition due to withdrawal or loss of animals during the study, one additional animal was included in each group, and six gilts were enrolled in each group of the study. General health indicators, intake of water and food, and behaviour were monitored daily. On the day of the first surgery (=day 0), pigs were pre-medicated (10 mg/kg BW ketamine, 0.5 mg/kg BW azaperone i.m.), anaesthetised (4 mg/kg Propofol i.v.) and intubated for isoflurane anaesthesia. The urine status was determined (Combur 10M, Roche, Basle, Switzerland). The urethra was examined by cystoscope (Obturator 27026 U, 17 Ch, 27005BA Hopkins II 0° optics, KarlStorz, Tuttlingen, Germany). The urethral wall pressure (UWP) was recorded by a urodynamics device (T-DOC 7Fr dual sensor, Nexam Pro WPU-L4; Laborie, Portsmouth, NH, USA; ≙UWP day 0 “pre”). Then, the gilts were put in deep anaesthesia (isoflurane 1 Vol% under controlled respiration). The sphincter deficiency was induced in two steps [25] by electro-dissection of the urethra (i.e., electrocautery, Vio300D, monopolar cut, effect 4, power maximum = 100 W; ERBE Elektrotechnik, Tübingen, Germany) and balloon dilatation as described [22,25,39]. Immediately after its induction, sufficient sphincter deficiency was confirmed by urodynamics as described above (≙UWP day 0 “post”). Randomisation or blinding were not applied.

### 2.6. Cell Therapy of Incontinent Gilts and Follow-Up

Sufficient sphincter deficiency was confirmed on day three in sedated gilts of the experimental cohort by urodynamics (≙UWP day 3) before cell injections, and the urine status was recorded. The MPCs (2.4 × 10^6^/mL in E-medium) of the male littermate were injected by Williams needle laterally (as it were 3 and 9 o’clock) in two aliquots of 250 µL each via transurethral route by cystoscope under visual observation [22,25]. During follow-up, the health status was monitored daily. From day seven onward, the urine status was recorded (Combur 10M, Roche, Basle, Switzerland), and sphincter deficiency and its recovery were monitored weekly for six weeks by urodynamics as described above. In the control cohort, SUI was induced and monitored as in the experimental cohort, but neither solvent nor MPCs were injected on day three of follow-up.

### 2.7. Statistics

The generated data were exported to Microsoft Excel for processing and subsequently transferred to GraphPad Prism (V.106.1) for statistical analysis. Continuous variables were summarised as means with standard deviations (SDs) or as medians with interquartile ranges (IQRs), depending on their distribution. Data normality was assessed using kurtosis, skewness, Q–Q plots, histograms, and the Shapiro–Wilk test.

The primary endpoint of the study was the urinary weight pressure (UWP) measured repeatedly in each animal over the six-week follow-up period. To evaluate longitudinal changes in recovery and differences between groups (control vs. therapy) over time, a linear mixed-effects model was fitted. The model included group, time (days), and their interaction as fixed effects. A random intercept for each animal was included to account for within-animal correlation due to repeated measurements. Models were fitted using restricted maximum likelihood (REML). Degrees of freedom were estimated using the Satterthwaite method. In case of significant group-by-time interaction, post hoc comparisons were performed by splitting the data by group. Tukey correction was applied for multiple testing, and statistical significance was set at *p* < 0.05.

To analyse the secondary outcomes of the *Merc’s* cell therapy, comparisons between two independent groups were performed using the independent samples *t*-test for continuous variables. Levene’s test was used to verify homogeneity of variance, and in cases where this assumption was not met, Welch’s *t*-test was applied. A 95% confidence interval and a *p*-value < 0.05 were considered statistically significant. Given the limited sample size, missing values were addressed using a single imputation approach to minimise data loss, preserve statistical power, and reduce potential bias associated with case-wise deletion.

## 3. Results

### 3.1. Production and Quality Management of Porcine Myogenic Progenitor Cells

**Figure 1:**
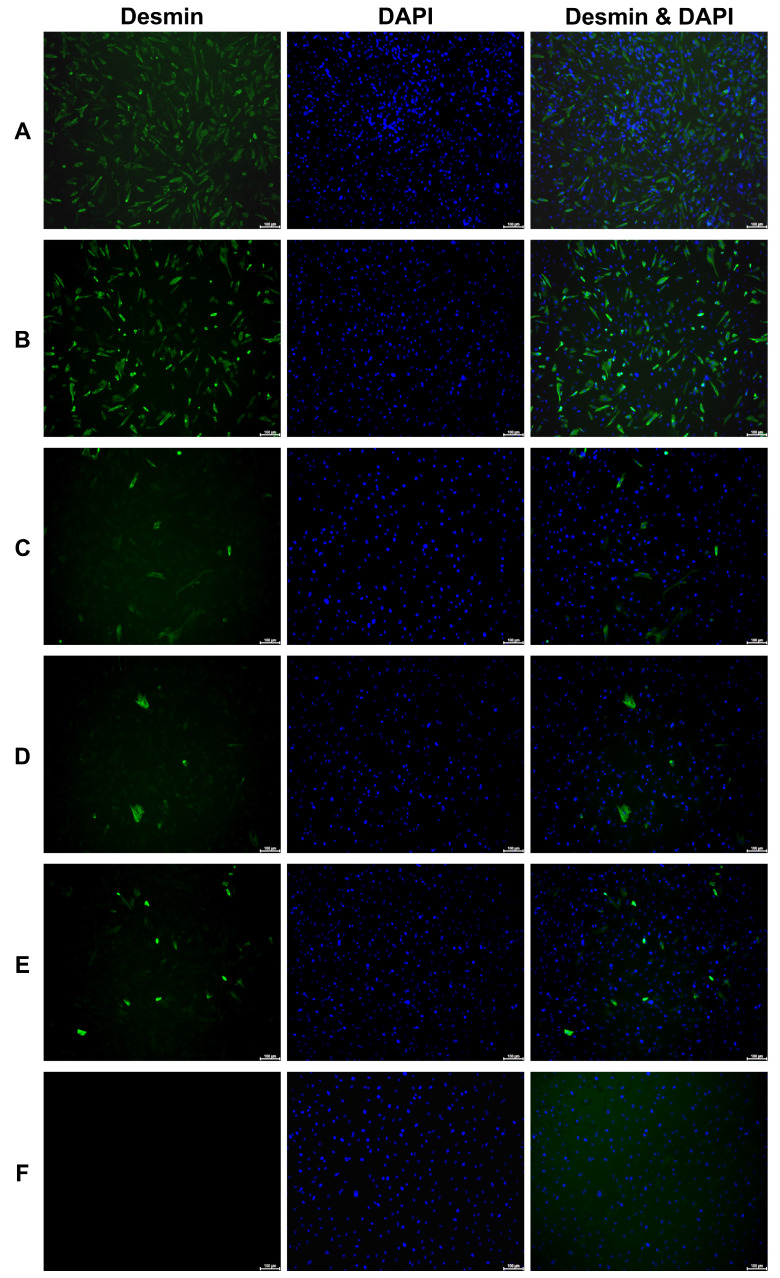
Detection of desmin expression on porcine myoblasts. Myoblasts were isolated from *M. extensor carpi radialis* (**A**,**B**), *M. rhomboideus cervicis* (**C**,**D**), and *M. fibularis tertius* (**E**,**F**) and expanded. The expression of desmin was visualised on adherent cells by ICC (green fluorescence). Cell nuclei were counterstained by DAPI (blue fluorescence). The fluorescence overlay is shown on the right panel. Faster cell proliferation and prominent desmin expression were recorded on *Mecr* MPCs (**A**,**B**). MPCs from *Mrc* and *Mft* expressed less desmin (**C**–**F**). The white size bars indicate 100 μm.

Myoblasts were isolated from striated muscle tissue containing elevated numbers of MPCs compared to the numbers of MPCs found in *M. semitendinosus* and used previously [28]. Accordingly, the MPCs were prepared from *M. extensor carpi radialis (Mecr)*, *M. rhomboideus cervicis (Mrc)*, and *M. fibularis tertius (Mft)*; expanded; and characterised. Cells isolated from *Mecr* expressed desmin at the highest levels and on most cells (Figure 1A,B) when compared to cells from the *Mrc* or *Mft* muscle, respectively (Figure 1C–F). Only minor differences in desmin expression between *Mecr* batch one (Figure 1A) versus *Mecr* batch two cells (Figure 1B) were observed. Comparably, major differences in desmin expression were not noted in batch one versus batch two of *Mrc* MPCs (Figure 1C,D). In contrast, the low desmin expression in batch one *Mft* MPCs was even undercut in batch two cells (Figure 1E,F). The data provided evidence for a higher yield of desmin-positive MPCs isolated from *Mecr* when compared to *Mrc* and *Mft* in pig muscle tissues [28]. The normalised desmin staining intensity index of *Merc* MPCs was 4.25, while *Mrc* MPCs (0.78) and *Mft* MPCs (0.95) scored below 1. This confirmed elevated desmin expression in *Merc* MPCs compared to the MPCs from the other muscles.

**Figure 2:**
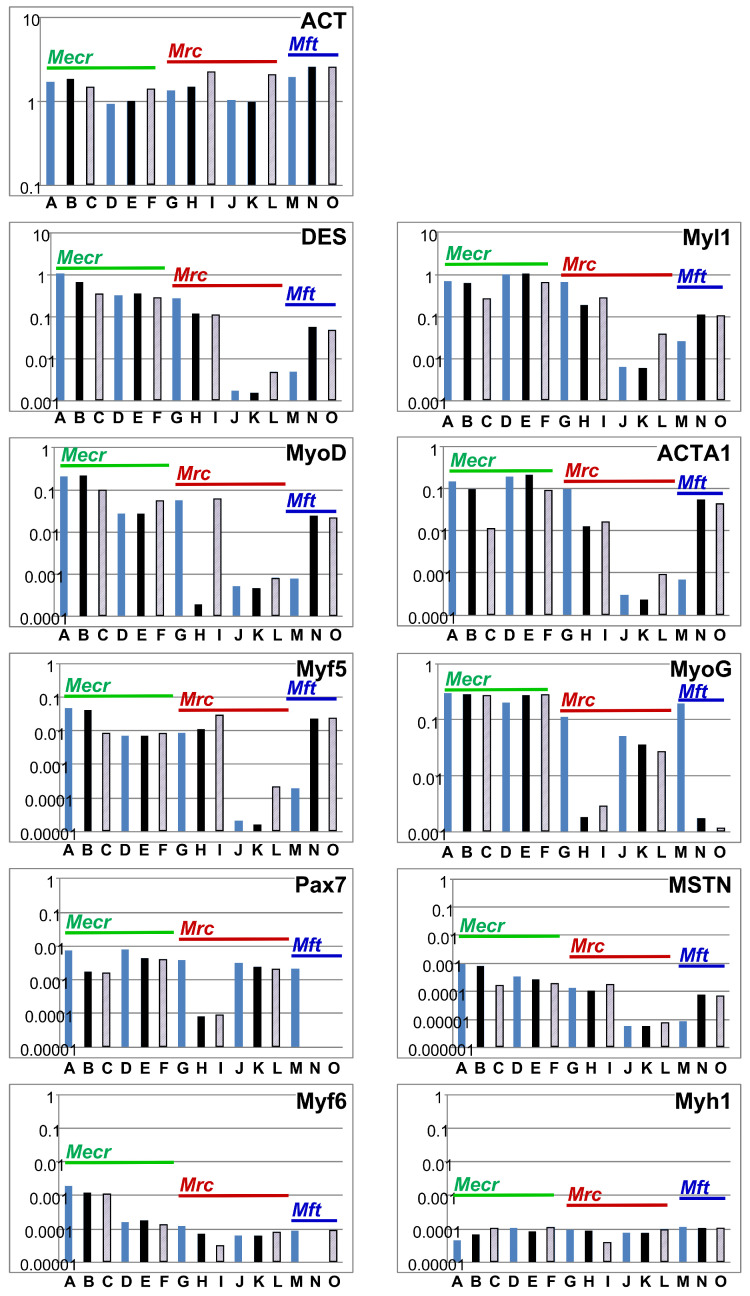
Expression of *β*-actin and myogenic marker genes by porcine myoblasts. Normalised transcripts encoding *β*-actin (**ACT**) and the myogenic factors (**DES**–**Myh1**) were enumerated in two different batches of MPCs: A–C: *Mecr* batch one, D–F: *Mecr* batch two. G–I: *Mrc* batch one, J–L: *Mrc* batch two. M–O: *Mft* MPCs. In addition, transcripts were enumerated in different passages: A, D, G, J, M: 2°P MPCs (light blue columns); B, E, H, K, N: 3°P MPCs prior differentiation (black columns); and: C, F, I, L, O: MPCs after differentiation in 3°P (grey dashed columns). In addition, the muscular source of MPCs is displayed in the individual graphs by a green line for *Mecr* MPCs, a red line for *Mrc* MPCs, and a dark blue line for *Mft* MPCs, respectively. The transcript expression of the individual genes, normalised to GAPDH, is shown on the *y*-axis of the diagrams, and the batches investigated on the *x*-axis.

**Figure A1:**
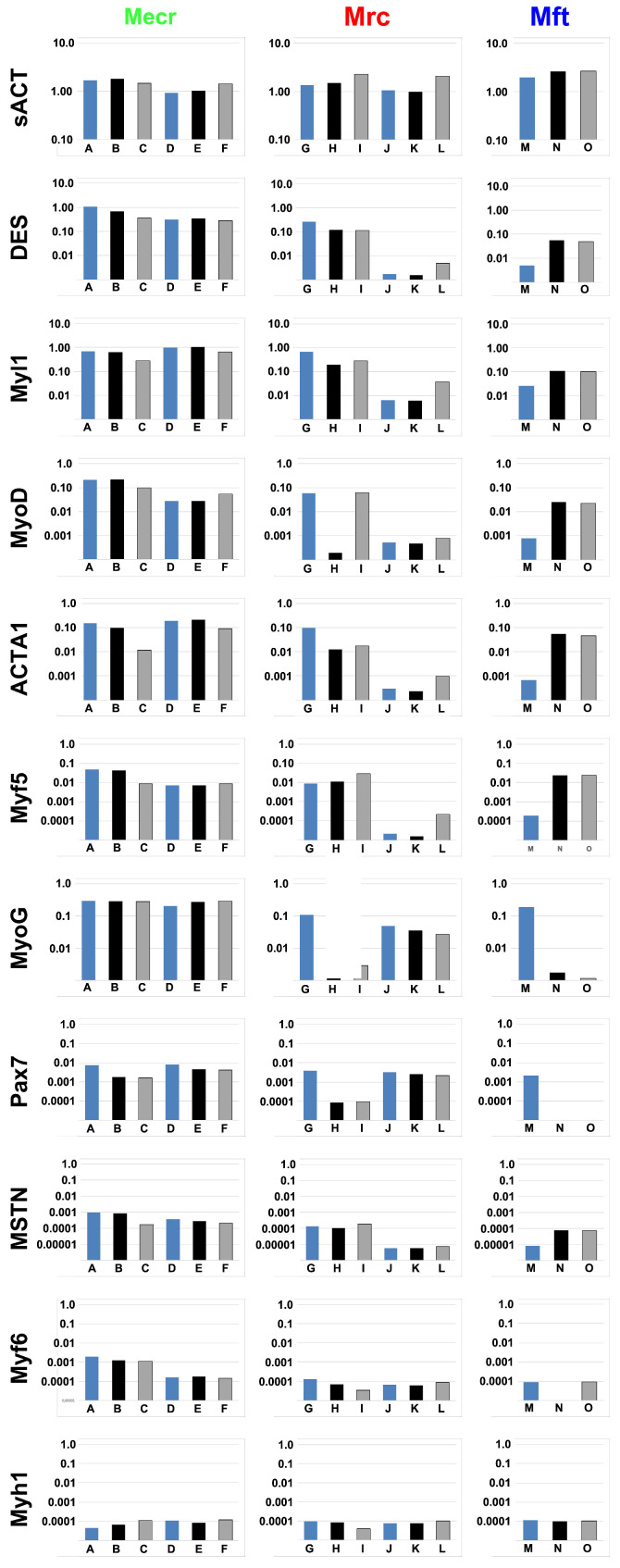
Expression of *β*-actin and myogenic marker genes by porcine myoblasts. Normalised transcripts encoding *β*-actin (ACT) and the myogenic factors (**DES**–**Myh1**) were enumerated in two different batches of *Merc* MPCs (left, columns A–F), *Mrc* MPCs (middle, columns G–H) and one batch from *Mft* MPCs (right, columns M–O). Light blue columns present the normalised transcripts of cells in the second passage, black columns present the normalised transcripts of cells in the third passage before induction of differentiation, and grey columns present the normalised transcript levels after induction of terminal differentiation in the third passage. The logarithm of the transcript expression of the individual genes, normalised to GAPDH, is shown on the *y*-axis of the diagrams, and the MPC batches A–O investigated on the *x*-axis. The sACT PCR was included in each run to ensure sufficient cDNA amounts in each batch for detection and quantification of the transcripts of the myogenic marker genes.

The expression of myogenic marker transcripts was investigated by RT-qPCR (Figure 2). In batches one and two of the *Mecr* MPCs, prominent transcript levels encoding desmin (DES) and myosin light chain-1 (Myl1) were detected. In *Mrc* MPCs batch two and in *Mft* MPCs, expression of DES and Myl1 were lower compared to *Mecr* MPCs (Figure 2). Transcripts encoding myogenic differentiation factor (MyoD), skeletal muscle actin alpha 1 (ACTA1), myogenic factor 5 (Myf5), myogenin (MyoG), as well as the early marker of MPCs paired box 7 (Pax7) were somewhat lower in all MPCs (Figure 2). Again, transcript levels of these factors were partially very low in batch two *Mrc* MPCs and in *Mft* MPCs (Figure 2). In addition, expression of myostatin (MSTN), myogenic factor 6 (Myf6), and myosin heavy chain-1 (Myh1) were explored and, overall, very low levels of transcripts were uncovered (Figure 2). The expression of Myf6, was elevated only in batch one MPCs from *Mecr*. The transcript analyses of the individual MPC batches in the second passage of in vitro culture can be depicted in the online supplement (Figure A1).

Furthermore, the expression of myogenic marker transcripts was investigated as a function of cell passage in 2°P MPCs (Figure 2 A, D, G, J, M; blue bars) versus 3°P MPCs before terminal differentiation (Figure 2 B, E, H, K, N; black bars) and after terminal differentiation (Figure 2 C, F, I, L, O, grey bars). Overall, batches one and two of *Mecr* MPCs presented promising and stable mRNA expression patterns throughout expansion and in vitro differentiation. Batch one *Mrc* MPCs expressed some markers, including DES, Myl1, ACTA1, and Myf5 in 2°P and 3°P cells at levels comparable to MPCs from *Mecr*, but MyoD, MyoG, and PAX-7 were very low in 3°P *Mrc* MPCs (Figure 2). Batch two of *Mrc* MPCs were excluded from cell therapy as Myl-1, MyoD, ACTA1, and Myf5 were found at low levels as well (Figure 2). In *Mft* MPCs, satellite cell marker Pax7 and myogenic progenitor cell marker MyoG were not detected in 3°P cells (Figure 2). Therefore, *Mft* MPCs were excluded from cell therapy for this reason as well. Myf6, a key factor associated with terminal differentiation of MPCs, was expressed at the highest levels in batch one *Mecr* MPCs. All other batches expressed this factor at considerably lower levels (Figure 2). The transcript levels of the individual MPCs in passage 3 before and after differentiation are disclosed online in Figure A1.

**Figure 3:**
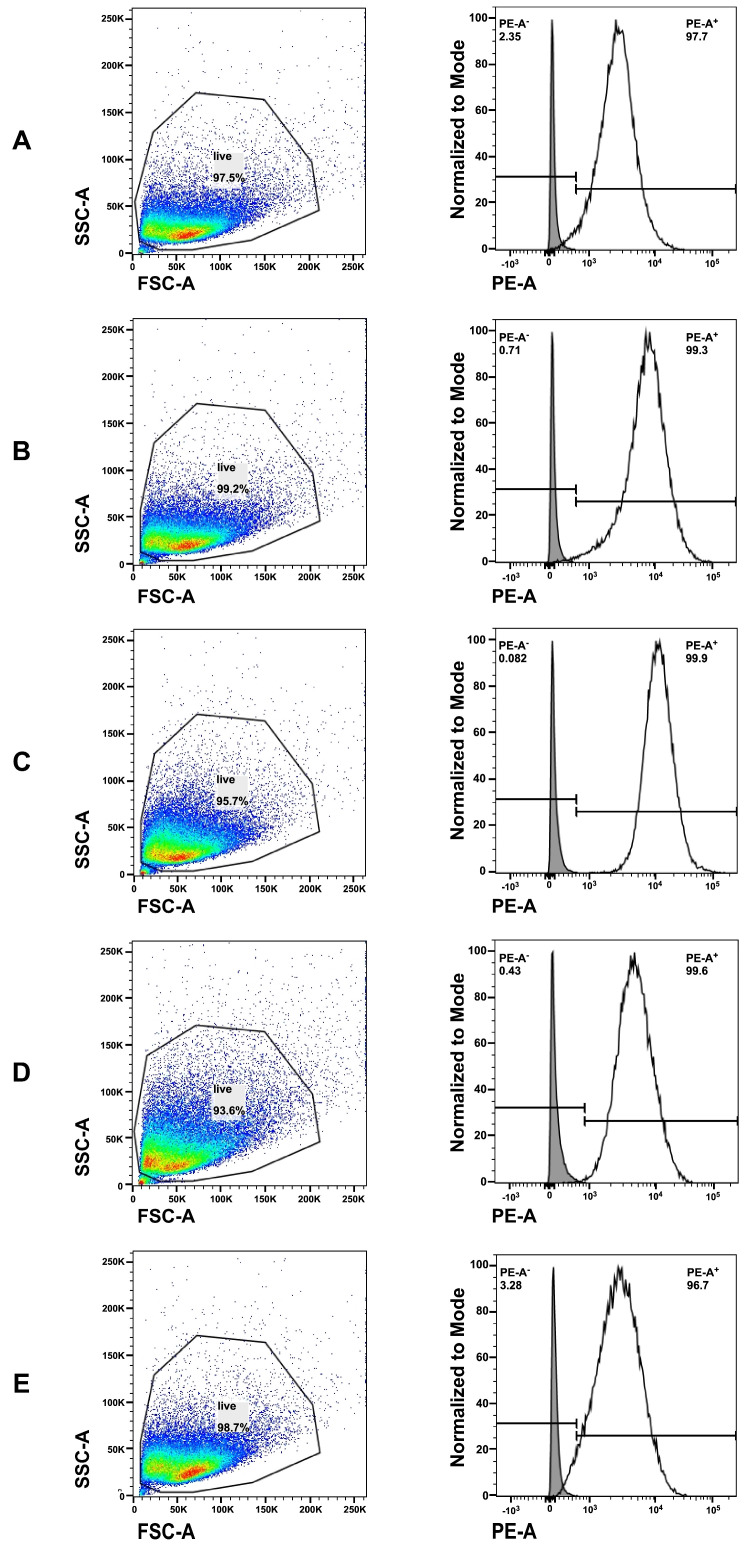
Detection of CD56 on porcine myoblasts by flow cytometry. Porcine myoblasts were isolated from *Mecr*, *Mrc*, and *Mft*, respectively, and the expression of CD56 was enumerated by FC. (**A**): *Mecr* batch one; (**B**): *Mecr* batch two; (**C**): *Mrc* batch one; (**D**): *Mrc* batch two; (**E**): *Mft MPCs*. Gating cell size (FSC-A) and granularity (SSC-A) facilitated the exclusion of dead cells and debris (left panel). The colours (red, yellow, green, blue) indicate the respective cell numbers detected at the corresponding forward- and side-scatters. The expression of CD56 was explored on live cells (right panel). The CD56 staining intensities are presented on the *x*-axis as histograms (white lines) compared to controls (grey histograms), and the number of the stained cells, normalised to mode, on the *y*-axis. The lines in the histograms indicate the range set for CD56-negative and positive cells.

CD56 is an adhesion molecule, and it is also considered a myogenic marker [34]. Thus, the CD56 expression was enumerated on the different MPC batches by flow cytometry (FC). More than 95% of the MPCs expressed CD56 with high mean fluorescence intensities. The narrow staining profiles of the histograms indicated uniform CD56-positive populations (Figure 3). This finding aligns with our recent studies [25,29].

**Figure 4:**
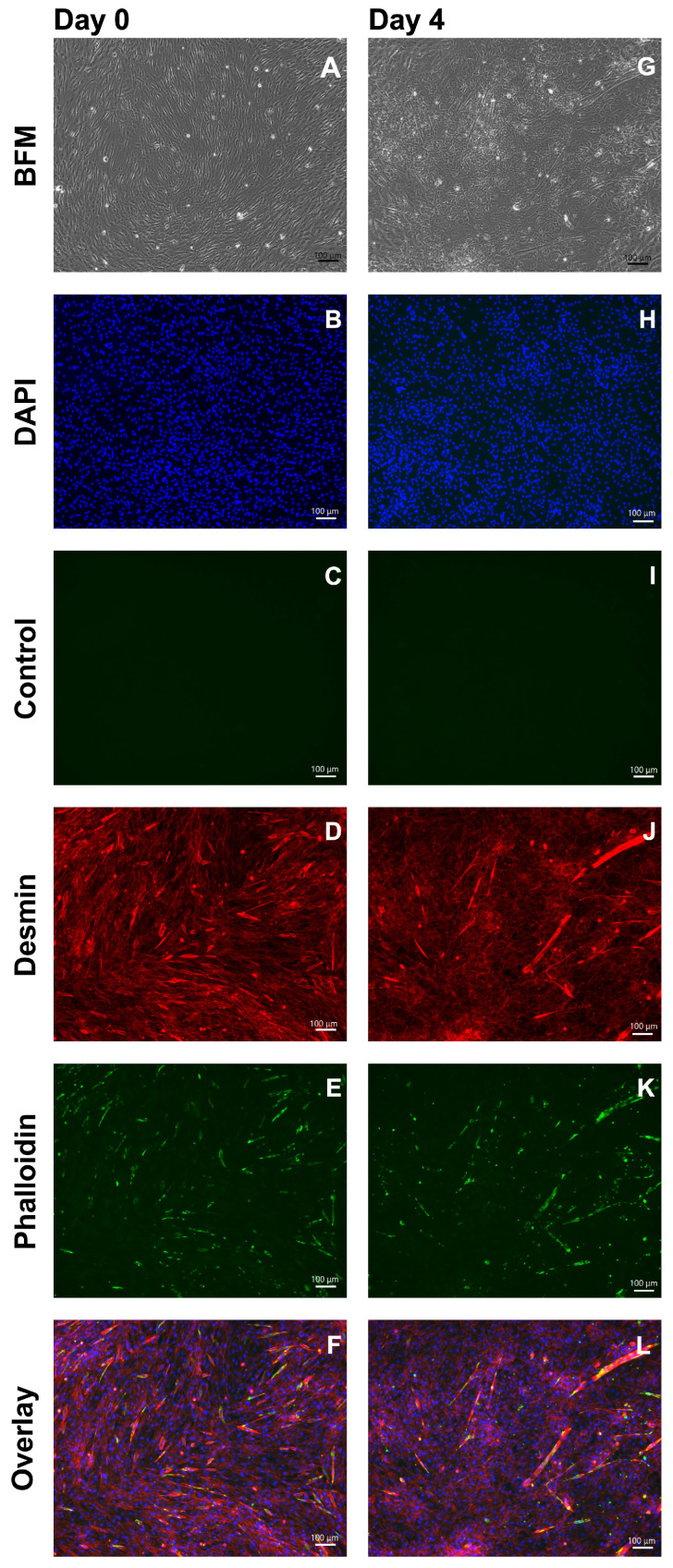
Terminal differentiation of *Mecr* MPCs to generate myofibres. The MPCs were seeded in chamber slides and expanded to confluence. MPCs incubated in E-medium served as controls (**A**–**F**). Terminal differentiation was induced, and the generation of myofibres was monitored by microscopy (**G**–**L**). Changes in the cell shapes were documented by brightfield microscopy (BFM; **A**,**G**), and the expression of desmin and f-actin by immunofluorescence as indicated (**D**–**L**). Cell nuclei were counterstained by DAPI (**B**–**L**), and samples omitting the primary anti-desmin antibody served as controls (**C**,**I**). Myotubes were not recorded in MPCs (**A**,**D**–**F**). By contrast, changes in cell shape were noted after differentiation by BFM (**G**) and the generation of elongated myotubes is documented by immunofluorescence employing the anti-desmin- and phalloidin staining (**J**,**K**) and in the overlay micrograph (**L**). Size bars indicate 100 μm.

**Figure 5:**
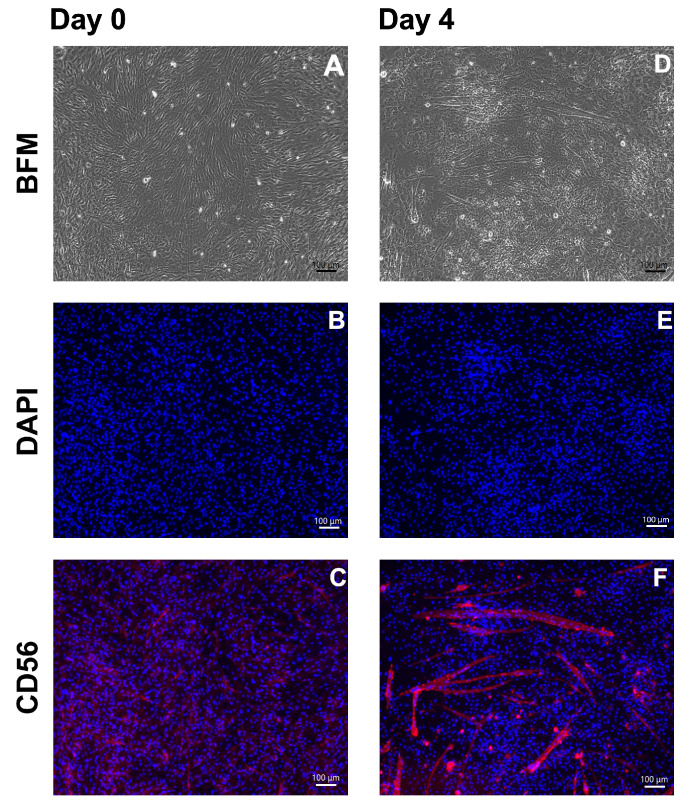
Expression of CD56 in *Mecr* MPCs and myotubes. The MPCs were seeded in chamber slides and expanded to confluence. Cells incubated in E-medium served as controls (**A**–**C**). Terminal differentiation was induced, and the generation of myofibres was monitored (**D**–**F**). Changes in the cell shapes were noted by brightfield microscopy (BFM; **A**,**D**). A bright expression of CD56 was noted on the elongated myotubes (**F**) when compared with the MPCs before differentiation (**C**). Cell nuclei were counterstained by DAPI (**B**–**E**). Size bars indicate 100 μm.

Terminal differentiation of MPCs and generation of myotubes are key to facilitating their integration in striated muscles during muscle tissue regeneration [40,41]. The terminal differentiation and generation of multinuclear myotubes were explored in pooled *Mecr* MPCs. The changes in cell shape and the generation of elongated multinuclear myofibres were recorded by microscopy and ICC (Figure 4 and Figure 5). Staining with anti-desmin antibodies and phalloidin (Figure 4) or with anti-CD56 antibodies (Figure 5) presented elongated myotubes after differentiation of the MPCs. Based on these analyses, *Mecr* MPCs were selected as candidate cells for the preclinical SUI cell therapy study.

### 3.2. Induction of Sphincter Deficiency in a Pig Model of Incontinence and Cell Therapy

**Figure 6:**
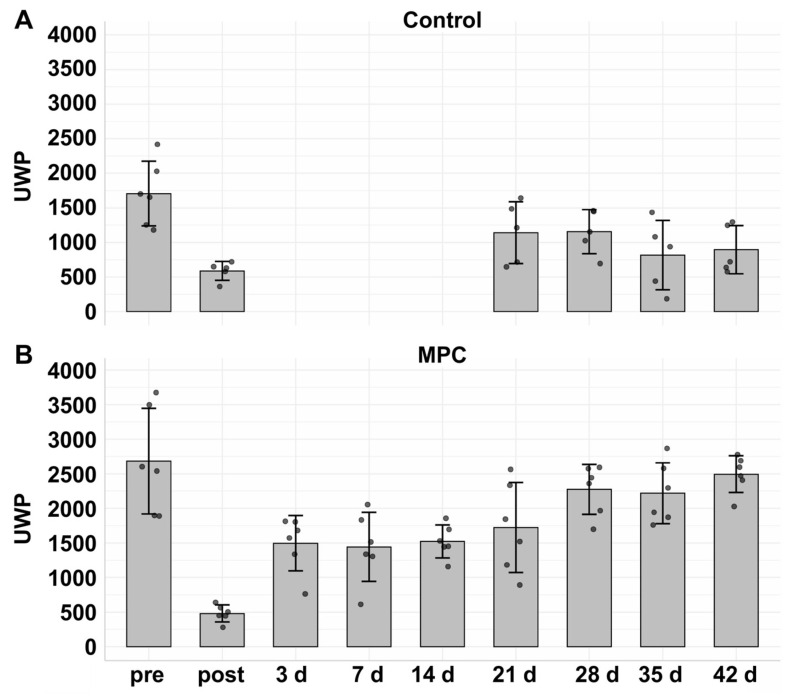
Induction of sphincter deficiency and functional recovery by cell therapy in gilts. The urethral wall pressure (UWP) was determined in untreated gilts (pre), immediately after induction of sphincter deficiency (post), and during follow-up as indicated on the *x*-axis. The y-axes display the mean UWP ± standard deviations in arbitrary units as indicated on the left and the normalised percentage of UWP relative to the starting point of untreated pigs (=100%) on the right side. (**A**) Mock-treated controls did not receive cell therapy. The spontaneous regeneration of the sphincter complex was monitored on days 21, 35, and 42 after induction of experimental UI, respectively. A serious drop in urethral wall pressure was recorded on day 0 immediately after its induction. The linear mixed model showed no significant functional recovery over time (β = 6.33, 95% CI −5.43 to 18.1, *p* = 0.304). (**B**) In the experimental cohort, sphincter deficiency was induced. This resulted in a serious drop in urethral wall pressure. This drop remained for three (3) weeks of follow-up, but on days 28, 35 and 42 an increase in UWP was observed. The linear mixed model showed that MPC-treated gilts recovered the sphincter function significantly during follow-up (β = 52.1, 95% CI: 40.4 to 63.7, *p* < 0.001). The urodynamics determined a full functional recovery (92%) compared to the starting point in MPC-treated gilts.

Sphincter deficiency was induced in the six gilts of the control cohort as described [22,25,39]. A robust sphincter deficiency was corroborated by urodynamics immediately after SUI induction in both groups (control cohort: 38.82% and experimental cohort: 19.3%) when compared to the percentage of UWP before SUI induction (=100%; Figure 6). Four weeks after sphincter injury, the spontaneous recovery reached 91.6% in the experimental cohort and 79.5% in the controls. To evaluate longitudinal changes in UWP recovery and differences between the therapy and control groups over time, a linear mixed model was performed. The model shows a significant group by time interaction, indicating that recovery trajectories differed between the groups over the four-week follow-up period (*p* value_interaction_ < 0.001). Therefore, a subgroup analysis was performed by stratifying the data according to group.

**Figure A2:**
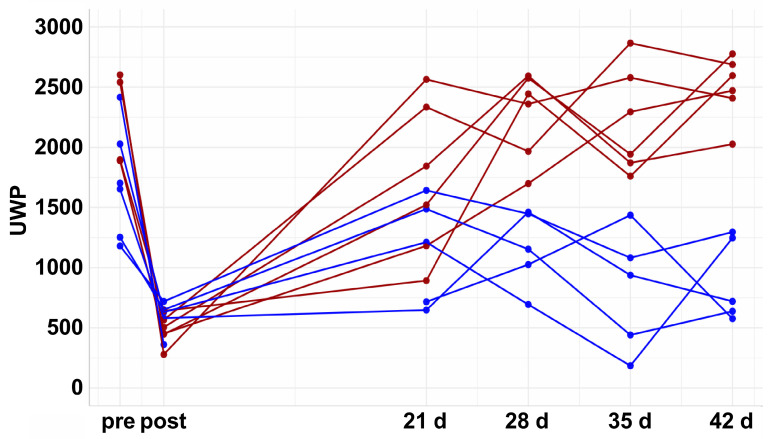
Induction of sphincter deficiency and functional recovery by cell therapy in the individual gilts. The urethral wall pressure (UWP) was determined in untreated gilts (pre), immediately after induction of sphincter deficiency (post), and during follow-up as indicated on the *x*-axis. The *y*-axis displays the normalised percentage of UWP relative to the starting point of untreated pigs (=100%). In the mock-treated controls (red lines), sphincter deficiency was induced, but the animals did not receive cell therapy on day three. In the experimental cohort (blue lines), sphincter deficiency was induced, and the pigs were treated by injection of *Merc* MPCs on day three. The graph presents the timelines of the UWPs of the individual pigs in the controls (red lines) versus the MPC-treated cohort (blue lines).

In the control group, an increase in the UWP after 21 days was recorded compared to the UWP immediately after SUI induction (“post”; Figure 6A). However, after 35 and 42 days of follow-up, the mean UWP dropped to 55.4% and 59.9%, respectively (Figure 6A). Longitudinal changes in UWP within the control group were assessed by using a linear mixed-effects model. No significant change in UWP was observed over time (β = 6.33, 95% CI −5.43 to 18.1, *p* = 0.304), suggesting that UWP does not improve over the follow-up period. The UWP timelines are presented online in Figure A2. These results are in line with our recent studies [25]. Due to a technical failure of the hardware for measuring urodynamics, UWPs could not be measured seven and fourteen days after SUI induction. Regrettably, a repetition of this control experiment was impossible. The animal welfare license did not allow for an additional six control animals, as our recent studies had already shown that the applied procedure yielded significant sphincter deficiency in landrace pigs (see [25,29]). We thus refer to these publications.

**Figure 7:**
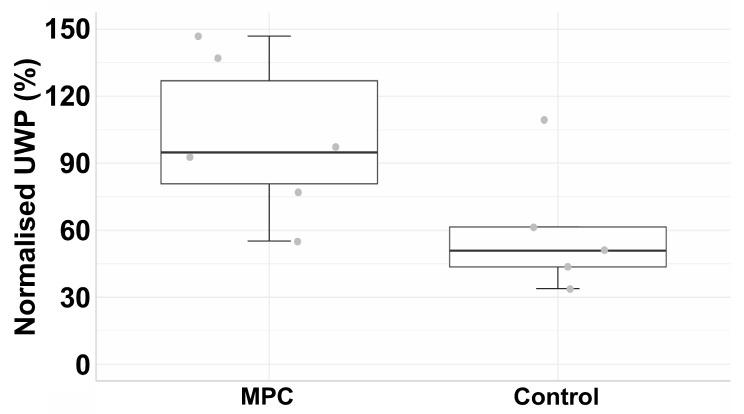
Outcome of the SUI cell therapy with *Mecr* MPCs. The figure illustrates a boxplot of urethral wall pressure stratified by group (MPC vs. mock control on the *x*-axis). The boxes represent the interquartile range (IQR), the horizontal lines in the boxes indicate the median, the whiskers show the range, and grey dots denote the mean value. The *y*-axis presents the normalised percentage of UWP relative to the starting point of untreated pigs (=100%).

In the experimental cohort, sphincter deficiency was induced in six gilts as described above. Immediately after SUI induction, sphincter deficiency was confirmed by urodynamics, and the UWP was reduced significantly to a mean level of 19.3% compared to levels before SUI induction (=100%) (Figure 6B). After urodynamics on day three, 1.2 × 10^6^ MPCs were injected by a Williams needle in the zone of electro-dissection. The UWP of MPC-treated pigs remained at levels between 56.7% and 64.9% for three weeks. However, a robust rise in mean UWP from 64.9% to 91.6% and 86.3% was noted on days 28 and 35, respectively (Figure 6B). The linear mixed model in the therapy group shows a significant increment in the UWP over time (β = 52.1, 95% CI: 40.4 to 63.7, *p* < 0.001; Figure 6B). Injection of MPCs facilitated an almost full and functional recovery to 92% compared to “post” (17%) (Figure 6B). After six weeks of follow-up, the UWP of the experimental cohort was significant compared to the mock controls (β = 1.32, 95% CI: 0.03 to 2.56, *p* < 0.045; Figure 7). The UWP timelines of the individual animals are presented in the supplement Figure A2. We conclude that, in contrast to our recent study employing MPCs from *M. semitendinosus* [25], MPCs from *M. extensor carpi radialis* facilitated a significant (*p* < 0.001) and almost full recovery (92%) of the urethral sphincter complex in the porcine large animal model of SUI (Figure 6 and Figure 7).

## 4. Discussion

The efficacy of cell therapy for urethral sphincter deficiency has been investigated in porcine SUI models. Yet, therapies using cells isolated from *M. gracilis* or *M. semitendinosus* were not convincing [25,42]. In contrast, injection of ADSCs yielded significant and full recovery of the sphincter complex in the same model [25]. Hence, the porcine large animal model of SUI seems suitable for investigating the efficacy of SUI therapies with different cells and distinct surgical technologies. In this context, we hypothesised that the MPCs applied in the two studies mentioned above [25,42] might have contributed to the outcome of these studies. Significant differences in the composition of muscle tissue and the blend of muscle precursor cells in distinct muscles are well known [43]. This includes differences in satellite cell and MPC densities, as well as differences in species, sex, and age of the donor [28,44,45,46,47,48]. Such differences may even depend on the applied cell culture protocols [28,29,49,50]. An analysis of Pax7^pos^ in muscular stem cells provided evidence that porcine *M. semitendinosus* contained fewer Pax7^pos^ muscle stem cells compared to, e.g., *M. psoas major* or *Mecr* [28]. The considerably low content of satellite cells and MPCs in *M. semitendinosus* could explain the insufficient regenerative potential in sphincter repair observed in our recent study [25]. Analogously, one would assume that MPCs from *Mecr* may have facilitated a superior performance and contributed to the significant and almost complete regeneration of the sphincter complex reported in this exploratory study.

Moreover, the composition of the sphincter muscle may influence the outcome of an MPC therapy of SUI: While in the human sphincter complex type-I fibre muscles predominate [51], in the porcine urethra type-II fibres prevail [52]. But one must not overlook that in the quadruped pig, striated muscles are predominantly localised in the distal part of the urethra [53,54], while the urodynamic peak is found in young female gilts more proximally [55]. In this zone, smooth muscle but not striated muscle builds the urethral closure complex in pigs [54]. Thus, the regeneration of the deficient sphincter by injection of MPCs most likely does not require integration and differentiation of the cells into the muscle but may be facilitated by the release of cytokines and growth factors. This, however, remains to be investigated in our future studies.

The published clinical SUI studies employed cells isolated from quite different muscles, including, for instance, cells from *M. quadriceps femoris* [56], *M. vastus lateralis* [20], *M. deltoideus* [57,58], *M. biceps brachii* [26,27], and *M. soleus* [17], or did not specifically mention the cell source. The rationale behind the choice of cell sources employed was often not disclosed, and the results reported in the clinical studies spanned from almost complete success to predominant failure. The different outcomes reported from the clinical SUI cell therapy studies might be associated, in part, with the different MPC populations employed. Moreover, the cells applied were not characterised in depth in all studies. This brings about additional complexity when comparing the outcomes of such studies. Despite all limitations, our results could suggest that successful SUI treatment of patients with muscle-derived cells might be more successful when using proliferation- and differentiation-competent MPCs. However, based on the data obtained in our porcine SUI animal model, we, at least, are wary of defining criteria for cellular or molecular quality measures of MPCs produced for SUI cell therapies on human patients. A meta-analysis of the results of previous SUI cell therapy studies combined with histological and/or cell biological analyses of the muscle tissue and MCPs used in the individual studies could provide some initial information on which myogenic markers might be relevant for therapeutic success in human SUI patients.

We selected the donor muscles for MPC production based on their content of progenitor cells [28], as MPCs can be expanded in vitro, while differentiated myotubes, and even more so myofibres, cannot be expanded in cultures under normal conditions. The differentiation of MPCs to myotubes was studied in vitro to investigate the phenotype of the *Merc* MPCs at this level. This does not imply that terminal differentiation of the injected MPCs and formation of myofibres in situ is key for sphincter regeneration in this model. Therefore, analyses of type-I or type-II myofibres in tissue samples ex vivo were not included after follow-up. Moreover, our recent studies provided evidence that injected cells cannot be tracked for such a long period of time [23,59]. Hence, our study falls short in providing robust evidence on the cellular processes of sphincter regeneration. Our proof-of-principle study suggests that, at least in the pig model of SUI, chances for better outcomes of sphincter regeneration are associated with MPCs isolated from a muscle enriched for satellite cells and/or myogenic progenitor cells.

In the current experiments, we did not plan a randomised controlled trial with large cohorts and multiple tissue donors per cohort. In contrast, this study focused on identifying more efficient MPCs for sphincter muscle regeneration. To this end, MPCs from different muscles were compared to the cells derived from *M. semitendinosus* used in our previous studies [22,25,38]. Pigs with induced sphincter deficiency without sham injections of interfering substances served as mock controls. The protocol for induction of sphincter deficiency, injection of MPCs, or urodynamics during follow-up was not altered in this study compared to our previous ones [25]. Nonetheless, future studies should be designed on a conformational level with larger cohorts and include additional controls such as a sham-injection of biologically inactive components. To avoid bias, such studies should be blinded with respect to verum versus placebo treatment and possibly even include independent teams or centres for cell production, animal surgery, and follow-up.

However, each preclinical animal model has specific disadvantages, and translating our results into clinical studies requires additional studies. In rabbits, for instance, regeneration of the urethral sphincter has been reported following the application of cells isolated from the *M. soleus* [60]. However, this regeneration was not superior to cell-free injection of the solvent, which contained plasma [60]. Moreover, the phenotype of the cells applied in this rabbit study was not investigated. One may conclude that the solvent complemented by plasma brought about sphincter regeneration in this set-up, not the cells. Thus, the efficacy of defined MPCs from *M. soleus* in sphincter regeneration remains to be investigated in animal models. By contrast, the application of MPCs from *M. soleus* in combination with electrophysiological stimulation seemed promising in women suffering from SUI [17]. Moreover, in most preclinical SUI animal studies, young animals were utilised. We noticed differences in the UWP profiles when young versus old as well as virgin versus multipara Göttingen minipigs were explored [55]. By contrast, most SUI patients are typically in an advanced stage of life. This necessitates a further restriction for the translation of our results in clinics. In addition, the individual breed of the pigs employed in such studies is critical when establishing an SUI model [37]. Reasons for these differences and the clinical relevance remain to be investigated.

Another factor contributing to the regenerative efficacy is the cell expansion protocol applied [28,29,49,50]. This includes the variables observed in the distinct cell sets or batches obtained in parallel preparations from the same muscle and donor animal reported here. For instance, batch one MPCs from *Merc* expressed higher levels of the early MPC marker MyoD than batch two MPCs from *Merc*. This is probably associated with the slightly different method utilised for the isolation of the cells. Likewise, different expression patterns between batches one and two were detected in MPCs from *Mrc* and *Mft* as well. An almost complete loss of the MPC-marker MyoG was reported in undifferentiated *Mft* MPCs over time [61], which is corroborated by our results from batch one MPCs from *Mrc* and *Mft*, but not from *Merc*. Changes in the expression of *β*-actin (ACT) as a function of cell culture passage or differentiation were not expected, as this protein is expressed in basically all nucleated cells. The consistently high expression levels of ACT demonstrated a comparable efficiency of RNA extraction, cDNA synthesis, and PCR amplification throughout the study.

The situation of the muscle to be targeted by MPC therapy may also contribute to the clinical success. Thus, in preclinical animal studies of SUI cell therapies, the injury of the urethra and the timeline until cell application will influence the outcome [41]. In addition, cell dosages investigated in animal studies may not translate directly to human therapies [14]. Moreover, the exact positioning of the injected cells contributes to the therapy’s outcome [24]. This, again, differs in humans in comparison, e.g., to the porcine SUI model due to the anatomical and physiological characteristics in these quadruped animals [22,53,62].

In our previous studies, a transient elevation in the UWP was recorded in the controls as well as in the verum cohort peaking approximately two weeks after SUI induction [24,25]. This data is missing for the control cohort in our study. Spontaneous muscle regeneration after mechanical injury will take about two weeks for measurable functional recovery [63]. Thus, this transient elevation in the UWP was associated with the spontaneous processes of sphincter repair after mechanical injury. But this was not the focus of the study. The focus of this study was to explore whether *Merc* MPCs facilitated better sphincter regeneration compared to the cells utilised previously [25,29].

## 5. Conclusions

Porcine myogenic progenitor cells isolated from *M. extensor carpi radialis* expressed high and stable levels of muscle stem cell markers Pax7 and Myf5, as well as myogenic progenitor markers MyoD and MyoG. Such cells facilitated a full and significant recovery from urethral sphincter deficiency in our large animal model of stress urinary incontinence. This study complements our recent publication, which provided evidence that ADSCs but not MPCs isolated from *M. semitendinosus* were capable of doing so. Thus, both types of cells, ADSCs as well as MPCs from *Mecr*, are promising candidates for further pre-clinical and possibly even clinical studies of cell therapy of incontinence.

## Figures and Tables

**Table 1:** Oligonucleotides for PCR of porcine myogenic marker transcripts and controls.

Gene	Forward Primer (5′->3′)	Reverse Primer (5′->3′)	Size (bp)
GAPDH	CCATCACCATCTTCCAGGAG	ACAGTCTTCTGGGTGGCAGT	346
ACT	CGGGCAGGTCATCACCATC	CGTGTTGGCGTAGAGGTCCTT	160
MyoG	CGCCATCCAGTACATCGAG	TGTGGGAACTGCATTCACTG	125
Pax7	AGATCGCAGCAGGGGTAAAG	GACCCCACCAAGCTGATTGA	209
Myl1	CTCTCAAGATCAAGCACTGCG	GCAGACACTTGGTTTGTGTGG	198
Myf5	GCTGCTGAGGGAACAGGTGGA	CTGCTGTTCTTTCGGGACCAGAC	135
MSTN	CCCGTCAAGACTCCTACAACA	CACATCAATGCTCTGCCAA	141
Myh1	CCAGGGAGAGATGGAGGACA	TCAAGTTCACGTACCCTGGC	258
Des *	ACACCTCAAGGATGAGATGGC	CAGGGCTTGTTTCTCGGAAG	176
Myf6	AGTGGCCAAGTGTTTCGGATC	CGCGAGTTATTTCTCCCCCA	179
ACTA1	ACCCGACGCCATGTGTGA	GTCGCCCACGTAGGAATCTT	184
MyoD *	CACTACAGCGGTGACTCAGACGCA	GACCGGGGTCGCTGGGCGCCTCGCT	145

* The temperature for primer extension was 60 °C for all primer pairs but Des and MyoD1. There, 62 °C was used.

## Data Availability

The original contributions presented in this study are included in the article. Further inquiries can be directed to the corresponding author.

## Abbreviations

The following abbreviations are used in this manuscript:

BFM	bright field microscopy
FC	flow cytometry
ICC	immunocytochemistry
*Mecr*	*M. extensor carpi radialis*
*Mft*	*M. fibularis tertius*
MFI	mean fluorescence intensity
MPC	myogenic progenitor cells
*Mrc*	*M. rhomboideus cervici*
SUI	stress urinary incontinence
UI	urinary incontinence
UWP	urethral wall pressure
